# The Prevalence of *Campylobacter* amongst a Free-Range Broiler Breeder Flock Was Primarily Affected by Flock Age

**DOI:** 10.1371/journal.pone.0022825

**Published:** 2011-12-12

**Authors:** Frances M. Colles, Noel D. McCarthy, Ruth Layton, Martin C. J. Maiden

**Affiliations:** 1 The Department of Zoology, University of Oxford, Oxford, United Kingdom; 2 The Food Animal Initiative, Wytham, Oxford, United Kingdom; Baylor College of Medicine, United States of America

## Abstract

*Campylobacter* successfully colonizes broiler chickens, but little is known about the longer term natural history of colonization, since most flocks are slaughtered at an immature age. In this study, the prevalence and genetic diversity of *Campylobacter* colonizing a single free-range broiler breeder flock was investigated over the course of a year. The age of the flock was the most important factor in determining both the prevalence and diversity of *Campylobacter* over time. There was no correlation with season, temperature, the amount of rain and sunshine, or the dynamics of colonization amongst geographically and temporally matched broiler flocks. The higher prevalence rates coincided with the age at which broiler chickens are typically slaughtered, but then in the absence of bio-security or other intervention methods, and despite changes in flock management, the prevalence fell to significantly lower levels for the remainder of the study. The genetic diversity of *Campylobacter* increased as the flock aged, implying that genotypes were accumulated within the flock and may persist for a long time. A better understanding of the ecology of *Campylobacter* within commercial chicken flocks will allow the design of more effective farm-based interventions.

## Introduction

*Campylobacter* is the most commonly reported bacterial cause of gastroenteritis worldwide, with *C. jejuni* causing the majority of infection and *C. coli* accounting for approximately ten percent of cases [1], [2]. Disease in industrialised countries is most often sporadic and the origin of infection difficult to trace since the organism can be isolated from the intestines of many animals and environmental sources including water [3], [4]. Contaminated poultry meat, however, is considered to be a major source of infection which may be partly due to the high bacterial load amongst live birds and the automation of the slaughter process [5], [6], [7], [8].

Typically, *Campylobacter* spreads rapidly through an intensively reared broiler flock within a matter of days and is rarely detected before two to three weeks of age [9]. Coprophagy and chicken to chicken transmission *via* the fecal-oral route increases the dissemination of the organism [9]. It is possible to reduce colonization of some housed flocks using strict bio-security measures, but *Campylobacter* can frequently be isolated from such flocks during transport, or from carcasses contaminated in the abattoir [10], [11]. It is not possible to use the same control measures for free-range and organically reared flocks, and high levels of contamination by both *C. jejuni* and *C. coli* have been recorded, often by multiple genotypes [9], [12]. Since most housed flocks that form the majority of the market in the UK are killed at five to six weeks of age, little is known about the course of infection above this age. Describing the natural history of infection in maturing chickens may improve our understanding of the host-colonizer relationship and guide interventions in younger birds.

The rates of colonization of broiler flocks increase during the summer months in temperate climates, although this can sometimes lag behind the peak in the incidence of human disease by a matter of weeks [4], [13]. The amount of sunshine and minimum temperature were found to be important factors correlating with broiler colonization in one study [13]. They may directly affect the transmission of the bacteria by varying the length of time the bacteria can survive outside the host, and also indirectly by influencing the presence and behaviour of mechanical vectors such as flies or wild birds [14]. It is also important to consider flock age, as well as seasonal effects, over a longer time period.

The aim of this study was to investigate the long term colonization of *Campylobacter* amongst a free-range broiler breeder flock kept over a year and compare the natural history of colonization over this time with that of immature broiler flocks. All isolates were sequence typed using seven locus MLST in order that the genetic diversity could be determined [15]. In addition, the effect of environmental parameters such as rainfall, temperature and sunshine were determined both directly, and indirectly by comparing *Campylobacter* prevalence and diversity with geographically and temporally matched broiler flocks.

## Results

### Prevalence of *Campylobacter*

**Figure 1:**
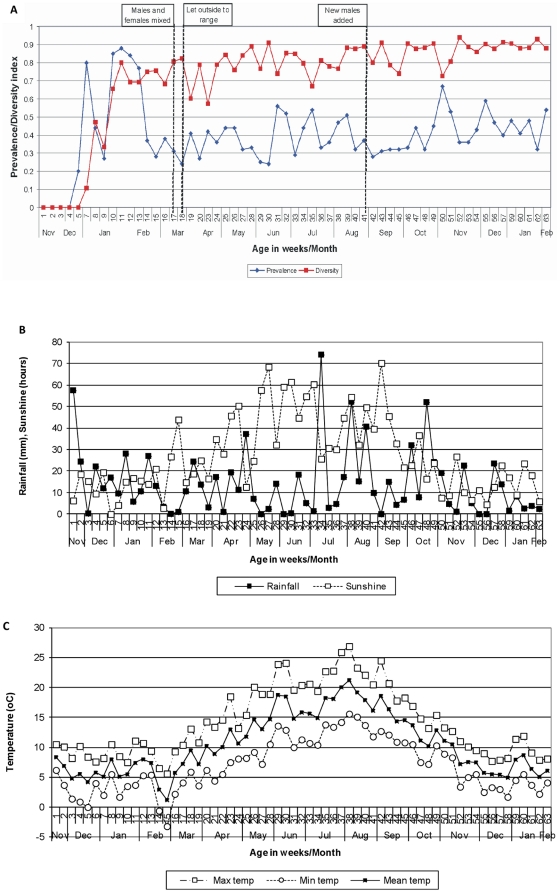
The prevalence and diversity index (A) of *Campylobacter* colonizing the free-range breeder flock over time. The weekly rainfall (mm) and sunshine (hours) (B) and the weekly maximum, minimum and mean temperature (°C) (C) recorded by the local Oxford Meteorological Office.

In the breeder flock, *Campylobacter* was first isolated from the feces of the five males that were tested at five weeks of age, but samples from 20 female birds tested were all negative, giving a prevalence rate of 0.2 (Fig. 1A). The birds were next sampled two weeks later when 80% of the birds tested, both male and female, were shedding *Campylobacter*. A peak in prevalence occurred between 10 and 13 weeks of age, with the highest rate of 0.88 occurring at 11 weeks (77 days) of age. The average prevalence rate for the period of five-13 weeks was 0.57. For the remaining weeks of the study the prevalence rate varied between 0.27 and 0.67, with an average rate of 0.40.

**Figure 2:**
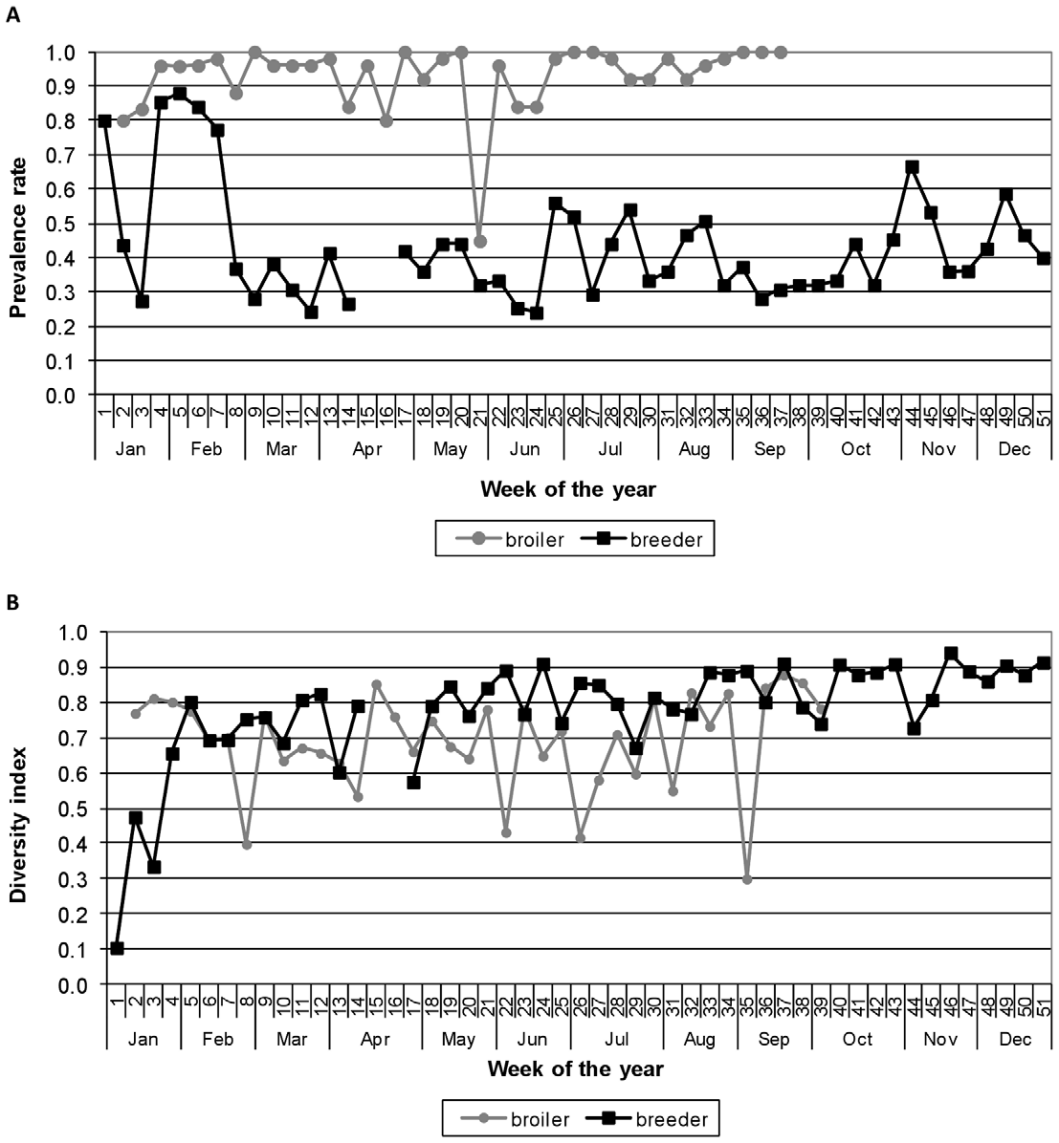
The prevalence (A) and genetic diversity (B) of *Campylobacter* isolated from the breeder and broiler flocks. The breeder flock was sampled over its life time of 63 weeks, whilst 37 geographically and temporally matched broiler flocks were each sampled at 56 days of age.

The prevalence rate of *Campylobacter* amongst the sequential broiler flocks each sampled at 8 weeks of age, ranged from 0.45-1 with an average rate of 0.91. For the flocks sampled between January and September in 2004, the year the two studies overlapped, the prevalence ranged from 0.45-1 with an average rate of 0.93 (Fig. 2A).

### Genetic diversity of *Campylobacter*

Initially the genetic diversity of *Campylobacter* genotypes was low, with the same genotype isolated from each of the male birds when they began shedding, and only two genotypes present when the flock was tested two and three weeks later (Fig. 1A). There was a rapid increase in diversity, averaging a ten percent gain in diversity per week, from when the flock first began shedding *Campylobacter* until the flock was 11 weeks of age (*D* = 0.8), and then a more steady increase over the rest of the study period, where the values varied between 0.57 (23 weeks of age) and 0.94 (52 weeks of age), and the average gain in diversity was 0.003 per week.

The diversity index of *Campylobacter* genotypes amongst the broiler flocks raised on the same farm in an overlapping time period and used for comparative purposes ranged from 0–0.95 with an average of 0.66. For the flocks sampled in 2004, the diversity index ranged from 0.30–0.88, with an average of 0.69 (Fig. 2B).

### Analysis of weather variables and changes in flock management

Following the initial sharp increase in *Campylobacter* prevalence up to week 13, and subsequent sharp fall at week 14, there was evidence that prevalence increased again slowly with time, but not that it varied seasonally (linear effect of time only, *p* = 0.024, R^2^ = 0.123, AdjR^2^ = 0.101; season and linear time, *p = *0.097, R^2^ = 0.1162, AdjR^2^ = 0.094). Similarly the genetic diversity of *Campylobacter* increased with time but did not show a seasonal pattern (linear effect of time only, *p*<0.001, R^2^ = 0.287, AdjR^2^ = 0.269; season and linear time *p* = 0.560, R^2^ = 0.287, AdjR^2^ = 0.268). There was no statistical support that the prevalence rate and genetic diversity of *Campylobacter* colonizing the breeder flock was related to that seen in the broiler flocks over the same time period using 1) predicted values from the broiler flock (prevalence, LR 1.48, *p* = 0.224; diversity, LR 0.08, *p* = 0.775) and 2) seasonal effects related to flock type (LR 5.4, *p* = 0.067). In addition, the results indicated that neither the prevalence rate nor genetic diversity of *Campylobacter* was influenced by the amount of rainfall, sunshine or temperature (Table 1, Fig. 1B and C). The *Campylobacter* prevalence over time was not affected by the birds being let onto the range (*p* = 0.193, R^2^ = 0.057) or by the addition of the new male birds to the flock (*p* = 0.351, R^2^ = 0.037). The genetic diversity continued to increase over time after the birds were let out (*p*<0.001, R^2^ = 0.564, average gain in diversity/week between weeks 18–40  =  0.016) and the new males added (*p*<0.001, R^2^ 0.589, average gain in diversity/week between weeks 41–63 = 0.009) but was consistent with forming an asymptote rather than the events having an impact.

**Table 1:** Modelling the effects of rainfall, sunshine and environmental temperature on the prevalence and diversity of *Campylobacter* genotypes colonizing a free-range broiler breeder flock.

	Meteorological variable	R^2^	*p* value
*Campylobacter*prevalence	Rainfall (mm)	0.012	0.408
	Sunshine (hrs)	0.039	0.136
	Maximum temperature (°C)	0.008	0.502
	Minimum temperature (°C)	0.000	0.879
	Mean temperature (°C)	0.002	0.754
*Campylobacter*diversity	Rainfall (mm)	0.002	0.772
	Sunshine (hrs)	0.028	0.225
	Maximum temperature (°C)	0.037	0.165
	Minimum temperature (°C)	0.058	0.079
	Mean temperature (°C)	0.047	0.117

## Discussion

As the majority of studies of *Campylobacter* prevalence have been conducted in housed broiler flocks that are typically slaughtered at an age of five to six weeks, there is little information concerning the long term colonisation of chickens with this organism. In addition, the methodology required presents technical challenges since it is not possible to take definitive samples from cecal contents on a longitudinal basis, and so it must be assumed that these results reflect the best estimate of dominant STs within birds that are present at levels detectable using swabs of the cloacal opening. Taking these limitations into account, the data collected in the present study provide evidence that in a single broiler breeder flock, the initial burst of infection that has been well documented amongst broiler flocks later fell to a significantly and consistently lower level in the absence of any intervention measures [9]. The maximum shedding rate coincided with the age range in which broiler chickens would typically be slaughtered. The sharp and largely sustained fall indicates the occurrence of an event that substantially controlled *Campylobacter* without any intervention.

The age of the flock was the most important factor in determining both the prevalence and genetic diversity of *Campylobacter* over time. The dynamics of initial flock colonization are thought to be age-dependent, potentially relating to changes in immunological maturity [9], and the difference in average prevalence seen in the 84 broiler flocks sampled at the single time point of eight weeks of age (0.91) and in the breeder flock over the course of 63 weeks (0.43) support this finding. The results are also in accordance with those from a pilot study on the same farm (unpublished data), when the average number of birds shedding *Campylobacter* between 77 and 112 days of age was half that recorded between 35 and 56 days of age. Continued influence of flock age on *Campylobacter* prevalence over a longer time period could additionally reflect physical differences in the mature bird and voided fecal matter and thus the apparent rate of prevalence, or the way in which the organism transfers between individuals. Other variables previously noted to influence *Campylobacter* infection in humans and poultry, such as weather and season, were tested directly, and also indirectly by comparing the fluctuations in the prevalence and genetic diversity with temporally and geographically matched broiler flocks, but none were found to show any correlation (Table 1) [13], [16]. These results support findings by Heuer *et al*, [17] who also found ‘within flock’ prevalence of *Campylobacter* to be unaffected by season. Changes in flock management that were necessary through the course of the study, such as mixing together different birds, and release onto the range, caused no discernable change in either prevalence or genetic diversity of *Campylobacter*. The results are consistent with those from a previous study on the farm indicating that environmental reservoirs of contamination such as wild birds and the range itself are not a major source of infection for free-range broiler chickens [18], [19]. Instead, genetic attribution studies indicate that many *Campylobacter* strains are host associated, and thus a more likely source of chicken associated genotypes is horizontal transmission from other chickens, for example *via* fomites contaminating equipment [20].

*Campylobacter* was isolated from the male birds at least one week before the females, despite being separated only by chicken wire with no bio-security measures. The male birds were nearer to the ark door and were also feed restricted which may have increased their susceptibility to infection [21], [22]. There is some evidence that males are slightly more susceptible in human disease [23], but there is no suggestion that the same is true amongst wild birds [13], [24]. Following the peak in *Campylobacter* shedding at 0.88 at 11 weeks of age, the average shedding rate fell to 0.40, similar to that of *C. jejuni* in wild Starlings (0.31) and wild geese (0.50) [24], [25], and may be the prevalence rate that is more typical of mature birds. Seasonal effects were detected amongst the wild birds but not amongst the breeder flock. This may be due to the heterogeneous nature of wild bird populations and significant differences in food availability throughout the year, in contrast to very closely related individuals within a poultry flock, that are fed a consistent diet.

The results were consistent with an initial bloom in colonization of very immature birds by a low number of genotypes (up to eight in this study), and with genotypes that were encountered later on being less dominant. The diversity of genotypes present in the flock increased rapidly, with a high rate occurring at the same time as the maximum rate in shedding. Unlike the shedding rate however, the index of diversity continued to increase, with the general trend forming an asymptote. Although the diversity of individual broiler flocks varied, the average at eight weeks of age, 0.66, was consistent with the lower diversity seen in the early weeks of the breeder flock, and evidence from a pilot study (unpublished data) also indicated that genetic diversity increased with flock age. The results suggest that the birds are exposed to an increasing number of *Campylobacter* genotypes as they age, and that they are accumulated within the flock rather than replacing other genotypes and being mutually exclusive. It is possible that some birds may become chronic shedders enabling genotypes to persist within the flock for a long time [26]. For *Campylobacter*, increased genetic diversity may promote its long term survival in the flock [27], [28]. The weekly fluctuations seen in the *Campylobacter* prevalence and genetic diversity may reflect the dynamics of infection associated with the introduction of different genotypes. High levels of genetic diversity (0.90–0.96) are evident amongst *C. jejuni* isolated from wild birds and so it would seem that this is a more natural situation in mature birds [24], [25].

In conclusion, with this particular free-range flock, bird age was more important than environmental parameters such as rainfall, temperature and hours of sunshine in determining both the prevalence and genetic diversity of *Campylobacter*. The peak in *Campylobacter* prevalence coincided with the age range in which broiler flocks are typically sent to slaughter and persisted for some six weeks, after which it declined and remained at a significantly lower level for the remainder of the flock life, without the need for intervention or bio-security. Further research is needed to determine whether these results are reproducible in other flocks and in other management regimes, and also whether the concentration of *Campylobacter* in feces changes as flocks age. If the naturally occurring drop at 14 weeks is consistent in other studies, it raises the question of whether the host factors responsible can be induced earlier as a component of *Campylobacter* control in the food chain, or whether it would be appropriate and financially viable in the interests of food safety to slaughter flocks later.

## Methods

### Ethical statement

We performed non invasive sampling that did not enter the body cavity of commercial birds and thus the need for approval under the Animals (Scientific Procedures) Act of 1986 was waived. The flock was a commercial broiler breeder flock and was slaughtered at 63 weeks of age at a commercial abattoir in line with standard industry practice. All prevailing local, national and international regulations and conventions, and normal scientific ethical practices have been respected.

### Chickens

The broiler breeder flock was the first raised on the commercially run Oxford University farm at Wytham, UK from November 2003 to February 2005. The birds were purchased as one day old chicks, 38 males (breed: Hubbard M77) and 500 females (breed: Hubbard JA57), and raised indoors until 18 weeks of age when they were let onto a fenced range during daylight hours. The males and females were initially kept in the same ark but separated by chicken wire due to the faster growing males being feed restricted; they were allowed together at 17 weeks of age. A group of 20 Hubbard Coloryield male birds were imported from France as full grown birds and kept in a separate location on the farm until they were mixed with the rest of the flock at 41 weeks of age. All of the 38 M77 male birds and 200 of the female birds were marked using numbered plastic leg rings (AC Hughes, Hampton Hill, UK) on each leg, between 14–16 weeks of age, and were used as a cohort within the main flock for more intensive study. The flock was slaughtered at 63 weeks of age.

*Campylobacter* isolates from 84 free-range broiler flocks (breed: Sherwood White in 2003 and Ross 308 females in 2004) raised on a rolling production cycle at the same farm site (with half moved to a second site 12 miles away at 24 days of age) were used for comparative purposes. In contrast to the breeder flock that was sampled longitudinally, each of the broiler flocks was sampled at a single time point shortly before slaughter at 56 days of age. Batches of 1,200 one day old broiler chicks were purchased and raised indoors until 24 days of age, when they were transferred to field arks and allowed onto a fenced range during day light hours at 28 days of age. The breeder and broiler flock studies overlapped during 2004, meaning that 36 of the broiler flocks were sampled at the same time points as the breeder flock.

### Bacterial culture and isolation

For the breeder flock, 25 fresh fecal samples were collected on a weekly basis from the birds between the ages of one and seven weeks (Table 2). After this time swabs of the cloacal opening were collected from 75 birds selected at random, and after 16 weeks of age, from 75 birds identified by leg rings and selected at random, provided they had not already been sampled that day. The male birds were not sampled after seven weeks of age as they became too difficult to handle. For the 84 broiler flocks, ten, 25 or 100 birds were selected at random and sampled for *Campylobacter* using swabs of the cloacal opening. The swab method was validated by comparison with samples of cecal contents from 19 culled birds from a separate broiler breeder flock at 57 weeks of age, matched by month (February), breed (Hubbard JA57) and farm site. Swabs gave an 88.9% sensitivity level, and were an acceptable proxy for *Campylobacter* populations recovered from cecal contents, although a greater number of swab samples were taken in this study due to their slightly lower recovery rate overall. The diversity of genotypes recovered from swab and cecal contents samples was indistinguishable (swab *D* = 0.90, CI 0.89–0.92, cecal contents *D* = 0.91, CI 0.90–0.93).

**Table 2:** The type and number of samples collected from the free-range broiler breeder flock over the course of the study.

Flock Age (Weeks)	Sample Type	Number of samples collected each week	Birds individually ringed
1 to 7	Feces	25	No
8 to 13	Swabs of cloacal opening	75	No
14 to 63	Swabs of cloacal opening	75	Yes

For all flocks, the fecal samples and swabs in charcoal transport media arrived at the laboratory within 1–2 of hours and were immediately cultured onto mCCDA (PO0119A Oxoid Ltd, Basingstoke, UK) and incubated at 42°C in a microaerobic atmosphere for 48 hours. Presumptive *Campylobacter* colonies identified by their typical colony type, positive catalase and oxidase reactions and gram negative curved rod morphology, were sub-cultured onto Columbia blood agar (PB0122A Oxoid Ltd, Basingstoke, UK) and incubated for a further 48 hours at 42°C in a microaerobic atmosphere. Chromosomal DNA was extracted from pure cultures either by boiling a cell suspension in PBS for ten minutes and removing the sediment by centrifugation at 13,000 rpm for five minutes, or by using commercial IsoQuick nucleic acid extraction kits (ISC Bioexpress, Kaysville, UT), following the manufacturer's instructions for the rapid DNA extraction protocol.

### Multi-locus and antigen sequence typing

The original protocol and reaction conditions for MLST were used, although the sequencing reactions were modified to 1/32 size reactions and a combination of the published primer sets was optimal [15], [29]. The short variable region (SVR) of the *flaA* locus was sequenced using the conditions and primers published previously [30], [31]. The nucleotide extension reaction products were detected on an ABI Prism 3730 automated DNA analyser and assembled using methods described previously [15]. The consensus sequence was queried against the *Campylobacter* database (http://pubmlst.org/campylobacter/) to give an allele number. The combination of alleles at the seven housekeeping genes used for MLST were assigned a sequence type (ST) and a clonal complex designation, if applicable, using the database. Clonal complexes consist of genotypes that share four or more alleles with the central genotype. Similarly the allele and peptide types for the *flaA* SVR sequences were assigned using the *Campylobacter flaA* database (http://pubmlst.org/campylobacter/flaA/).

### Meterological data

The meteorological data was supplied by the UK government Met Office (www.metoffice.gov.uk) and collected by the Oxford weather station.

### Statistical analyses

The prevalence of *Campylobacter* was calculated by dividing the number of positive samples by the total number of samples each week. A modified version of Simpson's index of diversity was used to calculate the diversity of *Campylobacter* genotypes each week, with a *D* value of 0 indicating that all individuals within a population are identical and a *D* value of 1 indicating that all individuals within a population are different [32]. The following formula was used:
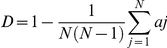
where *aj* is the number of types in the population which are indistinguishable from the *j*th strain, and *N* is the number of types in the population [32].

The effects of seasonality on *Campylobacter* prevalence and genetic diversity were tested for using harmonic linear regression analysis comparing the following variables, the linear effect of time V1 = week and the seasonal effect, the combination of V2 = sin(week/52×2π) and V3 = cos(week/52×2π). A seasonal effect was accepted as present if the adjusted *R^2^* value increased from that seen with the linear effect and there was statistical support (*p*<0.05) for the seasonal components of the model. Data from weeks one to fourteen reflecting the initial rise and peak in *Campylobacter* infection amongst the breeder flock were discounted from the analysis. The relationship between the prevalence and diversity of *Campylobacter* isolated from the breeder and broiler flocks that were collected at the same time points between weeks nine and 37 of 2004 was tested by 1) regression analysis with predicted fitted values from the broiler data over time and 2) regression analysis looking for seasonal effects (as above), with likelihood-ratio tests to determine the models of best fit. Regression analysis was also used to determine the effects of letting birds out onto the range, the addition of new males to the flock, and the amount of rainfall (mm), sunshine (hours), and minimum, maximum and mean temperatures (°C) on the prevalence and genetic diversity of *Campylobacter* colonizing the breeder flock. All statistical analysis was performed using the Stata data analysis and statistical software package (StataCorp LP, Texas, U.S.).
